# Synthesis and characterization of thiophene-derived palladium(ii) complex immobilized on FSM-16 and its application in the novel synthesis of 7-(aryl)-7,12-dihydro-6*H*-indeno[1,2,4]triazolo[1,5-*a*]pyrimidine-6-one derivatives†

**DOI:** 10.1039/d2ra06271b

**Published:** 2022-11-29

**Authors:** Azar Jahanbakhshi, Mahnaz Farahi, Bahador Karami, Iman Sedighimehr

**Affiliations:** Department of Chemistry, Yasouj University 75918-74831 Yasouj Iran farahimb@yu.ac.ir (+98)7412242167

## Abstract

The present study aims at synthesizing a palladium complex with a thiophene-carboimine ligand, supported on FSM-16 as a mesoporous silica support. Firstly, the prepared FSM-16 was modified using 3-aminopropyl group. The imine bond was subsequently formed by condensation of FSM-16-propyl amine with thiophene-2-carbaldehyde. Finally, the imine/thiophene-FSM-16 reacted with PdCl_2_ to form PdCl_2_-imine/thiophene-FSM-16. The structural and physicochemical properties of the prepared nanocomposite were characterized using FT-IR, TEM, XRD, FE-SEM, EDS, BET, and TGA analyses. PdCl_2_-imine/thiophene-FSM-16 exhibited efficient catalytic activity in the synthesis of indeno-1,2,4-triazolo[1,5-*a*]pyrimidine derivatives *via* a new three-component reaction between indan-1,3-dione, aromatic aldehydes and 3-amino-1*H*-1,2,4-triazole in water as the green solvent. Significantly, the heterogeneous catalyst can be easily separated from the reaction mixture and reused in another reaction.

## Abstract 2

The present study aims at synthesizing a palladium complex with a thiophene-carboimine ligand, supported on FSM-16 as a mesoporous silica support.

## Graphical abstract

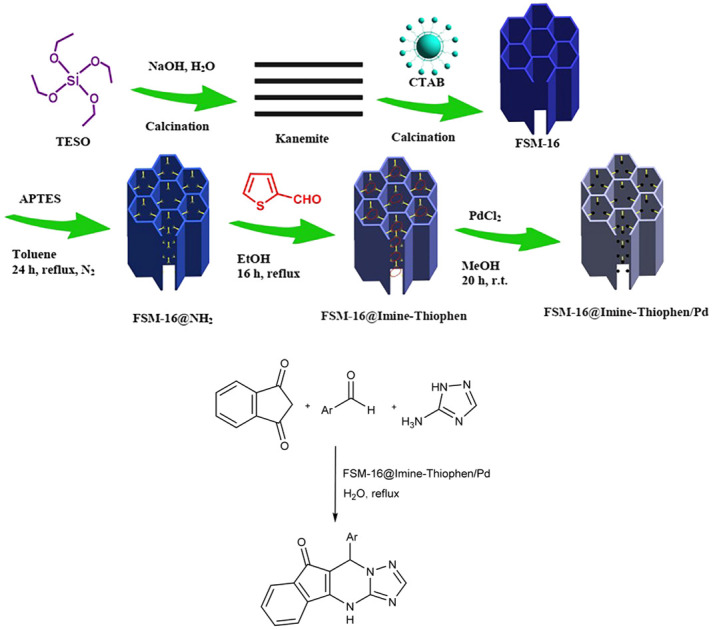

## 1. Introduction

In recent years, heterogeneous catalysts have attracted much attention due to simple separation, good stability, high performance, recyclability, and reusability.^1–3^ The heterogeneous catalysts with mesoporous structures have received intensive research due to their fascinating properties. Mesoporous silica materials with unique properties which possess high pore volume and surface area have also been regarded as significant compounds.^4–7^ They have pore sizes in the range of 2 to 50 nm.^8,9^ Mesoporous silica materials, such as FSM-16 and MCM-41, can be applied as solid substrates for adsorbents, chromatography, catalysts, and electrodes and also in drug delivery fields.^10–17^ Also, some important works reported on the synthesis of mesoporous Pd based nanocatalyst are Fe_3_O_4_@SiO_2_@IL-PMO/Pd, Fe_3_O_4_@MePMO-IL/Pd, HMS–CPTMS–Cy–Pd, MCM(Pd)-41.^18–21^ In this regard, FSM-16 has attracted great attention, as compared with other porous materials. Accordingly, a folded-sheet mechanism is applied, which results in forming a hexagonal array of channels.^22,23^ Because of the large surface area, large pore size, and high-density surface silanol sites, various functional groups can be grafted or incorporated onto the surface of FSM-16.^24,25^ Some transition metal complexes are prepared on the functionalized FSM-16 support and successfully used in organic reactions.^26–28^ Moreover, when the metal source is added to the kanemite preparation stages, various metals are directly substituted in the silica framework of FSM-16.^29^ FSM-16 shows thermal stability, which can be regarded as the most significant parameter in membrane application. Some recently studied FSM-16 catalysis systems are Fe_3_O_4_@FSM-16-SO_3_H, FSM-16/AEPC-SO_3_H, FSM-16-SO_3_H, and FSM-16-Met.^30–33^ Furthermore, FSM-16 materials have various uses in photo-metathesis reactions. Consequently, the development of synthetic procedures that yield novel heterogeneous FSM-16-based catalysts is still a great challenge for researchers.

Nowadays, the most critical challenge would be the design of effective chemical reaction sequences, providing great structural complexity with minimum synthetic steps.^34,35^ Multi-component reactions (MCRs) can be regarded as transformations in which, to form the product, we need more than two starting materials. Moreover, it is worth mentioning that such reactions will nearly incorporate the atoms of the starting materials. Multi-component one-step reactions play a significant role in synthesizing heterocyclic compounds. This is why the design of reactions based on these methods has received much attention for researchers in recent years.^36–38^ Heterocycles are among the most famous and remarkable compounds of organic chemistry and are also the basis of many drugs, chemicals, veterinarians, and agriculture.^39–41^ Many natural drugs such as atropine, codeine, reserpine, papaverine, and morphine are heterocycles.^42^ Also, new and efficient methods for synthesizing new heterocycles are still in high demand. The N-fused heterocyclic compounds, such as agrochemicals, colors, plastics and pharmaceuticals, are used in daily life. Amongst the best-selling therapeutic drugs, approximately one-third contain fused heterocyclic structures.^43–46^ Many powerful pharmaceutical products, biologically active compounds, and natural products contain bicyclic heterocycles fused with a pyrimidine.^47–52^ Among this plethora of combinations, the triazolo pyrimidines scaffolding was considered dramatically in natural compounds. Also, different triazolo pyrimidine derivatives have anti-inflammatory, antiviral, and anti-cancer properties. Triazolo pyrimidines have captured the attention of medicinal chemists owing to their wide variety of applications in medicinal chemistry and biochemistry.^53–56^

In recent years, multi-component reactions containing 1,3-indanedione have been successfully developed to synthesize spiro heterocycles, spirocycles, and fused heterocycles.^57–60^ The fused heterocycles are containing the 1,3-indanedione structure from important medicinal and biological frameworks. These compounds have attracted the attention of pharmacologists and chemists due to their broad scope of biological activities. Furthermore, they are widely used in materials, such as semiconductors and nonlinear optical properties of organic photovoltaic systems.^61–63^

**Scheme 1:**
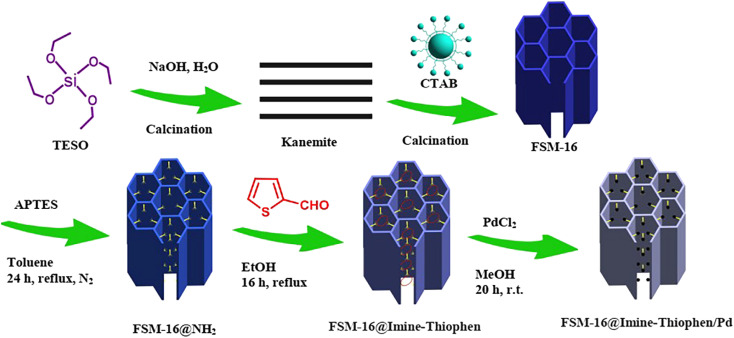
Synthesis of FSM-16@Imine-Thiophen/Pd nanocatalyst 1.

As a follow-up to our research program for the synthesis of new recyclable heterogeneous nanocatalysts,^64–71^ our attention was focused on FSM-16, which serves as a support for the immobilization of palladium complexes (Scheme 1). The FSM-16-based catalyst, also known as FSM-16@Imine-Thiophen/Pd, was used in the multi-component reactions of indan-1,3-dione, aromatic aldehydes and 3-amino-1*H*-1,2,4-triazole to produce indeno-1,2,4-triazolo[1,5-*a*]pyrimidine derivatives.

## 2. Experimental

### 2.1. Materials

The reagents, chemicals and solvents including tetraethyl ortho silicate (TEOS), cetyltrimethyl ammonium bromide (CTAB), sodium hydroxide (NaOH), 3-aminopropyltriethoxysilane (APTES), thiophene-2-carbaldehyde, PdCl_2_, 3-amino-1*H*-1,2,4-triazole, indan-1,3-dione, benzaldehyde, 4-nitrobenzaldehyde, 4-chlorobenzaldehyde, 2-methylbenzaldehyde, 3-ethoxy-4-hydroxyphenylbenzaldehyde, 4-bromobenzaldehyde, indole-3-carboxaldehyde, 4-(benzyloxy)benzaldehyde, ethanol, toluene, methanol were purchased from Fluka, Merck, and Aldrich-Sigma and applied without further purification.

### 2.2. Instrumental measurements

The FT-IR spectra were carried out using FT-IR JASCO-Model 680 spectroscopy area of 400–4000 cm^−1^. The NMR spectra analyses were done using Bruker 400 MHz Ultrashield spectrometer device at 400 MHz (^1^H NMR) and 100 MHz (^13^C NMR) in DMSO-*d*_6_ as solvent. The energy-dispersive X-ray (EDX) analysis was obtained by applying the TESCAN vega model. The nanoparticles morphology was measured using FE-SEM-TESCAN MIRA3, and X-ray analysis was done using the following diffractometer: Rigaku Ultima IV. TEM images were taken using Philips EM208S, the Netherlands, TEM apparatus at 100 kV. Brunauer–Emmett–Teller (BET) determines the technique used to analyze the surface area. Thermogravimetric (TGA) analysis was done using a Perkin-Elmer-6000.

### 2.3. Catalytic tests

#### 2.3.1. Preparation of FSM-16@NH_2_

FSM-16 was synthesized based on the literature method.^25^ Then, the prepared FSM-16 (0.5 g) was dispersed in dry toluene (15 mL) for 15 min, and then 3-aminopropyltriethoxysilane (APTES) (3 mL) was added dropwise, and the reaction mixture was stirred at 90 °C for 24 h under N_2_ atmosphere. The resulting solid was filtered, washed with dry toluene and dried.

#### 2.3.2. Synthesis of FSM-16@Imine-Thiophen

In the following, the FSM-16-NH_2_ (0.5 g) was dispersed in ethanol (20 mL) by sonication for 20 min, and thiophene-2-carbaldehyde (1 mL) was added to the mixture. The mixture reaction was stirred for 16 h at 80 °C under an N_2_ atmosphere. The solid product (FSM-16@Imine-Thiophen) was filtered, washed several times with ethanol and dried.

#### 2.3.3. Preparation of FSM-16@Imine-Thiophen/Pd (nanocatalyst 1)

FSM-16@Imine-Thiophen (0.5 g) was dispersed in MeOH (30 mL) for 30 min, and then PdCl_2_ (0.17 g) was added, and stirred at room temperature for 20 h. In the end, the resultant solid was separated, washed with MeOH, and dried.

#### 2.3.4. General procedure for the synthesis of indeno[1,2,4]triazolo[1,5-*a*]pyrimidine 5

A mixture of indan-1,3-dione (1 mmol), aldehyde (1 mmol), 3-amino-1*H*-1,2,4-triazole (1 mmol) and nanocatalyst 1 (0.007 g) was refluxed in H_2_O (3 mL). TLC was applied to monitor the progress of the reaction. Subsequently, ethanol (10 mL) was added, and the catalyst was separated by filtration. Finally, the pure product was obtained by recrystallization from EtOH.

#### 2.3.5. Spectral data

##### 2.3.5.1 7-Phenyl-7,12-dihydro-6*H*-indeno[1,2,4]triazolo[1,5-*a*]pyrimidine (5a)

FT-IR (KBr) (*ν̄*_max_, cm^−1^): 3163, 3062, 2958, 2888, 1708, 1623, 1591, 1530, 1460, 1330, 1309. ^1^H NMR (400 MHz, DMSO-*d*_6_): *δ* = 11.3 (s, 1H), 8.68 (s, 1H), 8.16 (d, 1H, *J* = 7.6 Hz), 7.83 (t, 2H, *J* = 7.2 Hz), 7.09 (d, 2H, *J* = 7.6 Hz), 6.91 (t, 2H, *J* = 8.8 Hz), 6.80 (t, 2H, *J* = 7.2 Hz), 5.89 (s, 1H). ^13^C NMR (100 MHz, DMSO-*d*_6_): *δ* = 188.76, 167.13, 156.01, 150.14, 144.71, 142.78, 141.20, 139.16, 135.46, 133.40, 130.10, 125.67, 118.84, 109.96, 105.86, 35.16.

##### 2.3.5.2 7-(4-Bromophenyl)-7,12-dihydro-6*H*-indeno [1,2,4]triazolo[1,5-*a*]pyrimidine (5b)

FT-IR (KBr) (*ν̄*_max_, cm^−1^): 3188, 3107, 3085, 2911, 2855, 1716, 1623, 1590, 1528, 844 670, 523. ^1^H NMR (400 MHz, DMSO-*d*_6_): *δ* = 12.10 (s, 1H), 8.08 (d, 1H, *J* = 7.6 Hz), 7.63 (t, 1H, *J* = 6.4 Hz), 7.58 (t, 1H, *J* = 7.4 Hz), 7.50 (d, 2H, *J* = 8 Hz), 7.36 (d, 2H, *J* = 8 Hz), 7.15 (d, 2H, *J* = 6.8 Hz), 7.03 (s, 1H), 5.69 (s, 1H). ^13^C NMR (100 MHz, DMSO-*d*_6_): *δ* = 190.52, 165.81, 153.95, 139.86, 136.27, 135.19, 134.67, 132.26, 131.97, 130.73, 127.62, 123.92, 123.07, 122.79, 117.32, 33.76.

##### 2.3.5.3 7-(4-Nitrophenyl)-7,12-dihydro-6*H*-indeno[1,2,4]triazolo[1,5-*a*]pyrimidine (5d)

FT-IR (KBr) (*ν̄*_max_, cm^−1^): 3136, 3062, 2923, 2851, 1731, 1618, 1589, 1423, 1326, 1309. ^1^H NMR (400 MHz, DMSO-*d*_6_): *δ* = 11.15 (s, 1H), 8.17 (d, 2H, *J* = 8 Hz), 8.12 d (d, 2H, *J* = 8.4 Hz), 8.07 (d, 2H, *J* = 7.6 Hz), 7.83–7.93 (m, 2H), 7.71 (t, 1H, *J* = 7.2 Hz), 5.92 (s, 1H). ^13^C NMR (100 MHz, DMSO-*d*_6_): *δ* = 188.59, 166.95, 157.40, 152.36, 146.58, 142.29, 137.41, 137.08, 133.24, 132.90, 129.53, 125.12, 117.33, 111.15, 104.50, 36.01.

##### 2.3.5.4 7-(1*H*-Indol-3-yl)-7,12-dihydro-6*H*-indeno[1,2,4]triazolo[1,5-*a*]pyrimidine (5e)

FT-IR (KBr) (*ν̄*_max_, cm^−1^): 3178, 3459, 3147, 2960, 2844, 1708, 1612, 1588, 1401. ^1^H NMR (400 MHz, DMSO-*d*_6_): *δ* = 11.32 (s, 1H), 11.11 (s, 1H), 7.49 (d, 1H, *J* = 8.8 Hz), 7.38 (t, 2H, *J* = 8.4 Hz), 7.31 (s, 1H), 7.13 (d, 1H, *J* = 6.8 Hz), 7.01 (t, 2H, *J* = 6.4 Hz), 6.60 (d, 2H, *J* = 7.2 Hz), 6.49 (s, 1H), 5.12 (s, 1H). ^13^C NMR (100 MHz, DMSO-*d*_6_): *δ* = 190.17, 162.31, 154.17, 151.40, 140.99, 137.12, 129.92, 129.58, 128.60, 128.36, 127.90, 125.59, 122.58, 118.24, 116.64, 113.44, 111.06, 34.65.

##### 2.3.5.5 7-(3-Ethoxy-4-hydroxyphenyl)-7,12-dihydro-6*H*-indeno[1,2,4]triazolo[1,5-*a*]pyrimidine (5h)

FT-IR (KBr) (*ν̄*_max_, cm^−1^): 3336, 3274, 3119, 2941, 2876, 1725, 1622, 1549, 1438. ^1^H NMR (400 MHz, DMSO-*d*_6_): *δ* = 12.17 (s, 1H), 11.11 (s, 1H) 7.80 (d, 1H, *J* = 8 Hz), 7.51 (t, 1H, *J* = 7.6 Hz), 7.33 (t, 2H, *J* = 7.2 Hz), 7.15 (s, 1H), 6.83 (s, 1H), 6.75 (d, 2H, *J* = 8 Hz), 6.64 (d, 1H, *J* = 8 Hz), 5.05 (s, 1H), 3.58 (q, 4H, *J* = 7.6 Hz), 1.86 (t, 3H, *J* = 7.6 Hz). ^13^C NMR (100 MHz, DMSO-*d*_6_): *δ* = 188.90, 168.35, 161.59, 160.27, 159.53, 155.62, 153.59, 139.27, 135.53, 135.27, 128.38, 117.17, 114.32, 108.83, 107.09, 103.83, 101.35, 65.37, 35.31, 26.13.

## 3. Results and discussion

### 3.1. Synthesis and characterization of the catalyst

Herein, the synthesis and characterization of the novel FSM-16-supported nanocatalyst 1 is reported, following the protocol shown in Scheme 1. To prepare FSM-16@Imine-Thiophen/Pd, FSM-16 was primarily synthesized and functionalized with APTES to obtain FSM-16-NH_2_ nanostructure. Afterward, FSM-16-NH_2_ reacted with thiophene-2-carbaldehyde to synthesize FSM-16@Imine-Thiophen. Subsequently, regarding the metal coordination complex, PdCl_2_ was used, affording FSM-16@Imine-Thiophen/Pd nanocatalyst. The synthesized nanocatalyst was investigated using XRD, FT-IR, TGA, FE-SEM, EDS, TEM, and BET techniques.

**Fig. 1:**
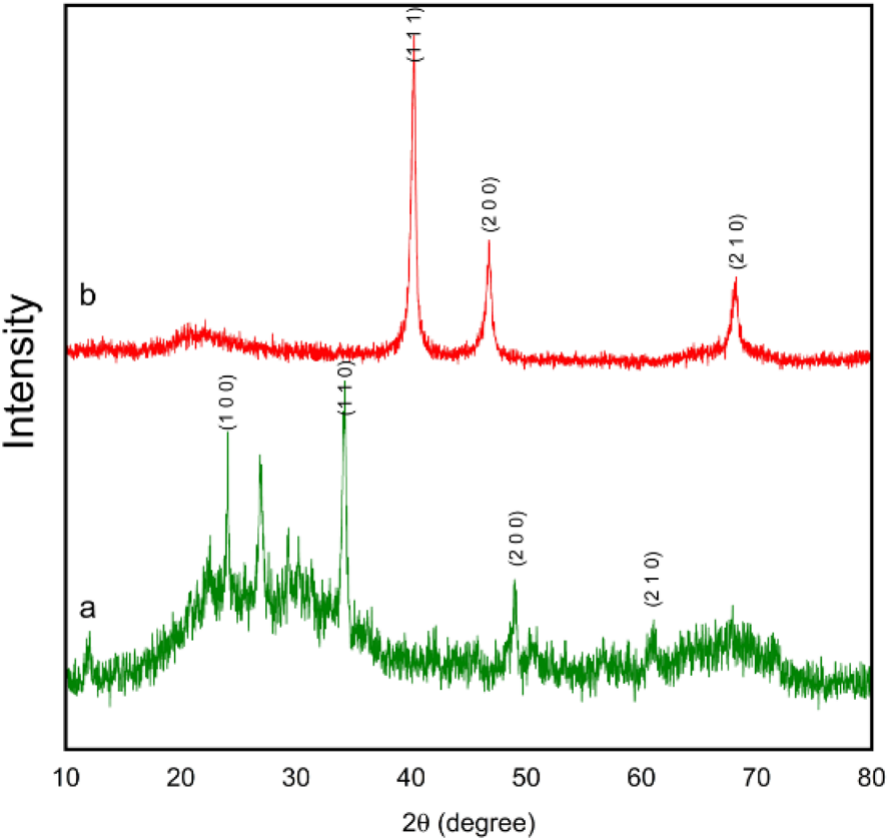
XRD patterns of FSM-16 (a) and FSM-16@Imine-Thiophen/Pd nanocatalyst (b).

The TEM, FE-SEM, and XRD analyses of FSM-16@Imine-Thiophen/Pd nanocatalyst were performed to get insight into the structural features of the material. As Fig. 1 illustrates, XRD diffraction patterns of FSM-16 and FSM-16@Imine-Thiophen/Pd catalyst are shown in a range of 2*θ* = 10–80°. As shown in Fig. 1a, XRD patterns of FSM-16 exhibit four peaks indicative of ordered hexagonal mesostructures at 2*θ* = 23.4°, 37.1°, 48.5°, and 61.5° assigned to the (100), (110), (200) and (210) planes.^72–76^ Accordingly, three new peaks can be observed at 2*θ* = 39.5°, 45.4°, and 68.5°, corresponding to (111), (200), and (220) planes, respectively, which signify the presence of palladium complex on the functionalized FSM-16.^75,76^ Furthermore, as indicated in Fig. 1b, the intensity of the principal peaks are decreased as compared to bare FSM-16, which is due to the immobilization of organic groups and Pd complex on FSM-16 pore walls.

**Fig. 2:**
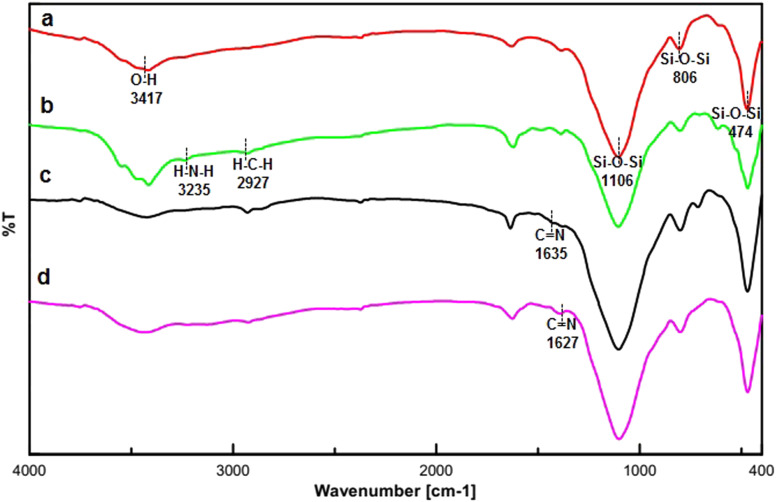
The FT-IR spectrum of FSM-16 (a), FSM-16@NH_2_ (b), FSM-16@Imine-Thiophen (c) and FSM-16@Imine-Thiophen/Pd (d).

The FT-IR spectra of FSM-16, FSM-16-NH_2_, FSM-16@Imine-Thiophen, and FSM-16@Imine-Thiophen/Pd are illustrated in Fig. 2. Fig. 2a indicates a stretching vibration of the –OH group at about 3417 cm^−1^. The bands at 474 cm^−1^, 806 cm^−1^, and 1106 cm^−1^ signify the symmetric and asymmetric vibrations of the siloxane group (Si–O–Si). Regarding the spectra of FSM-16-NH_2_ (Fig. 2b), the appearance of the new peaks at 2927 cm^−1^ and 3235 cm^−1^ belong to CH_2_ and NH_2_, respectively, which confirms the presence of APTES on FSM-16. Thus, the disappeared of the 3235 cm^−1^ peak relevant to the NH_2_ bonds affirms the formation of FSM-16@Imine-Thiophen. Furthermore, as Fig. 2c shows, the band at 1635 cm^−1^ is related to the C=N bond. According to Fig. 2d, the shifted imine band to a lower frequency in the FSM-16@Imine-Thiophen/Pd nanocatalyst (from 1635 cm^−1^ to 1627 cm^−1^), which confirms the formation of palladium complex on the surface of FSM-16@Imine-Thiophen.^11,31–33^

**Fig. 3:**
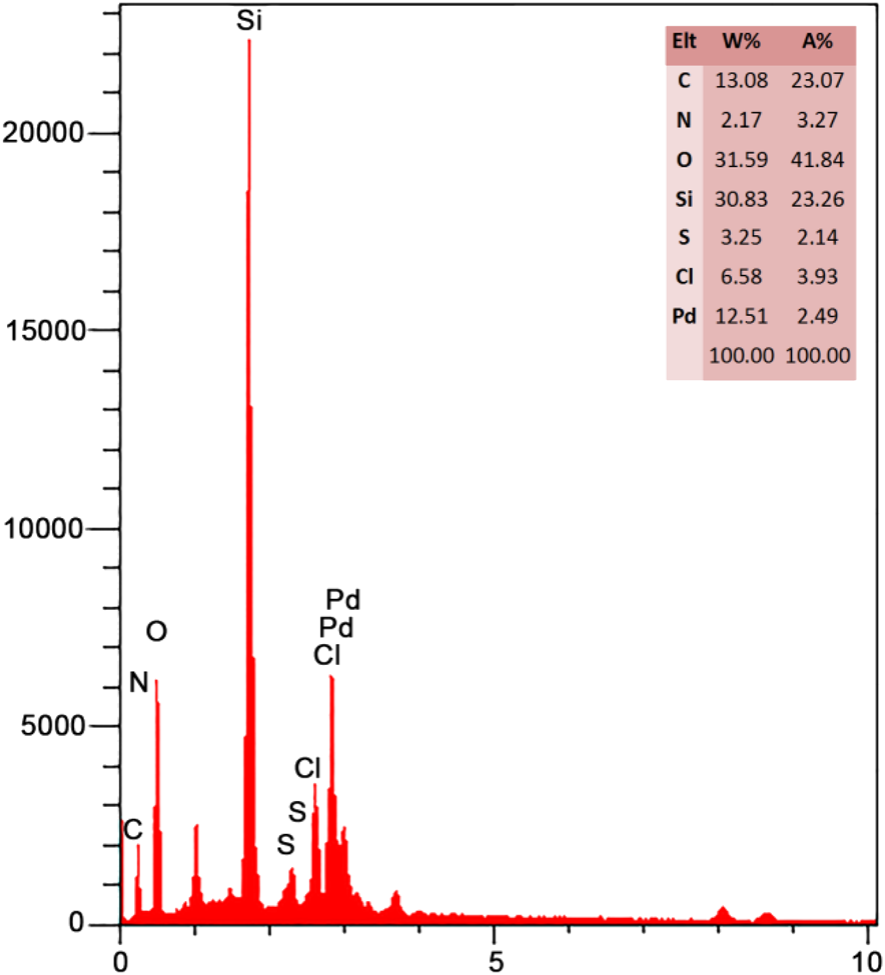
EDX spectra of FSM-16@Imine-Thiophen/Pd nanocatalyst.

According to Fig. 3, the EDX spectrum of FSM-16@Imine-Thiophen/Pd contains all expected elemental cases, including C, O, N, S, Si, Pd, and Cl elements.

**Fig. 4:**
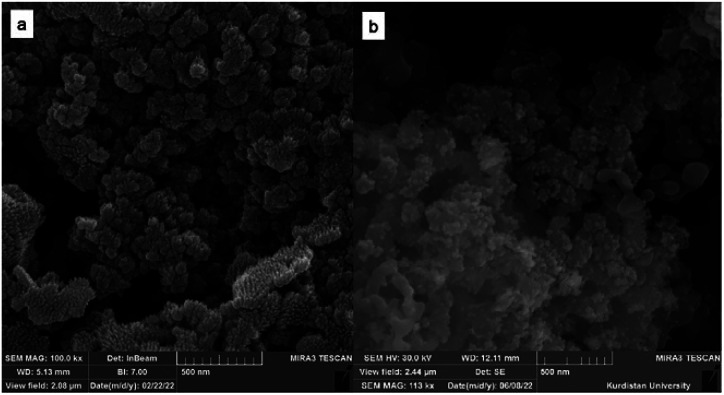
The FE-SEM images of FSM-16 (a) and FSM-16@Imine-Thiophen/Pd (b).

The characterization of morphology and size of FSM-16 and FSM-16@Imine-Thiophen/Pd nanoparticles was performed using field effect scanning electron microscopy (FE-SEM) (Fig. 4). Based on FE-SEM images, the surface morphology of the catalyst shows spherical particles, with a regular size of less than 100 nm, possessing a highly ordered pore structure. Furthermore, the compounds FSM-16 and FSM-16@Imine-Thiophen/Pd have similar structures, and there are no significant differences.

**Fig. 5:**
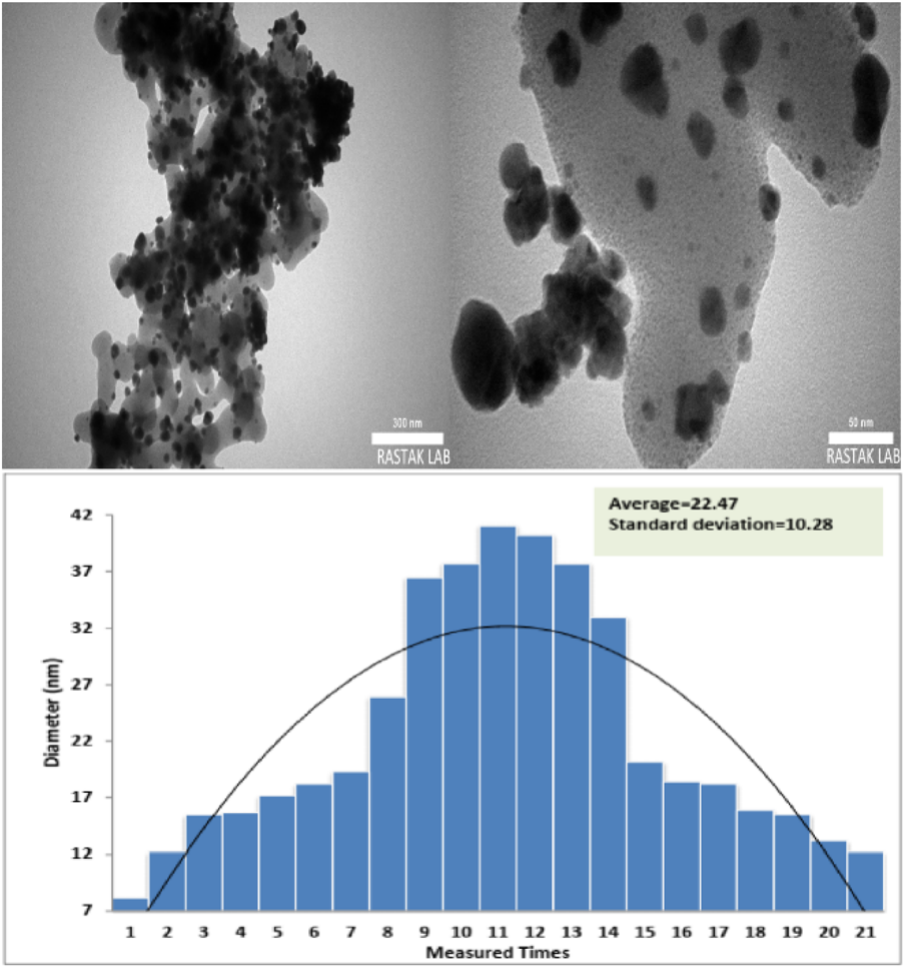
TEM image of FSM-16@Imine-Thiophen/Pd.

Herein, TEM images of FSM-16@Imine-Thiophen/Pd nanocatalyst, which are shown in Fig. 5, indicate the hexagonal channels with uniform pore size. In these images, the presence Pd nanoparticles with black color on the gray layer FSM-16@Imine-Thiophen have appeared. The histogram of the particle size distribution of the prepared nanocatalyst showed the dimensions of the distribution in the range of 7 to 39 nm and with a mean size of 23 nm.

**Fig. 6:**
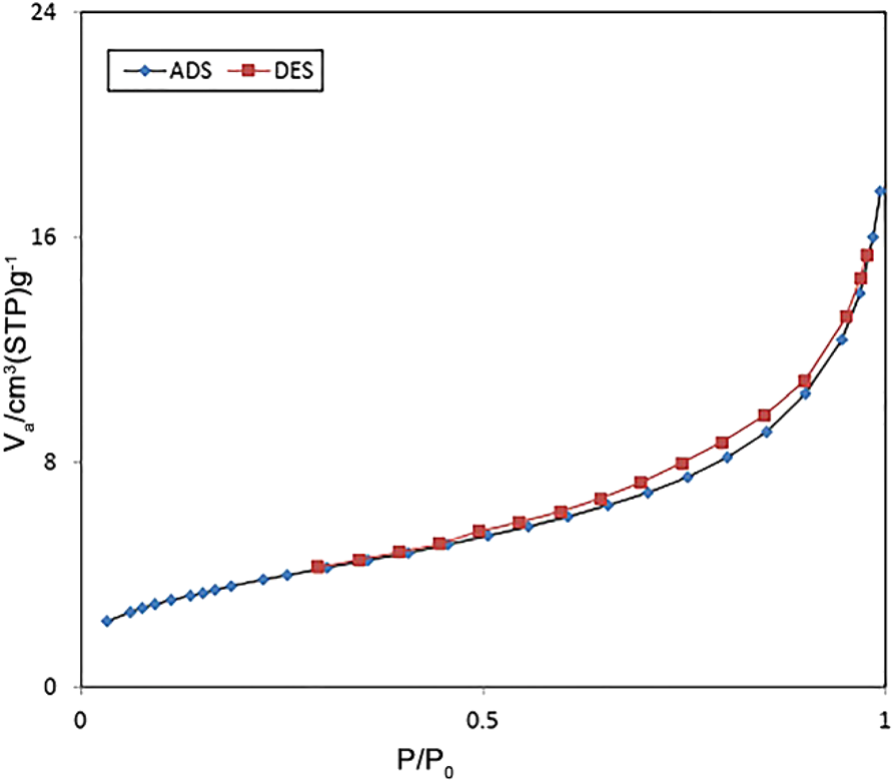
Nitrogen adsorption–desorption isotherms of FSM-16@Imine-Thiophen/Pd nanocatalyst.

Fig. 6 indicates the nitrogen adsorption–desorption isotherms of FSM-16@Imine-Thiophen/Pd nanocatalyst. According to the IUPAC classification, the resulting catalyst is a type VI isotherm, which suggests the presence of mesoporous structures. Based on the BET analysis, the total pore volume, surface area, and mean pore diameter of the catalyst were obtained at 136 m^2^ g^−1^, 0.02 cm^3^ g^−1^, and 7.75 nm, respectively.

**Fig. 7:**
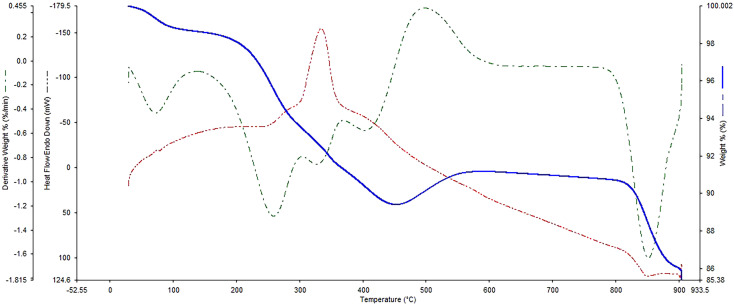
TGA analysis of the FSM-16@Imine-Thiophen/Pd nanocatalyst.

Fig. 7 illustrates the TGA analysis of FSM-16@Imine-Thiophen/Pd, which was done to examine the thermal stability of the FSM-16@Imine-Thiophen/Pd nanocatalyst. Regarding the TGA curve, the first weight loss occurs at a temperature below 200 °C of about 2% due to the removal of hydroxyl groups and the physically and chemically adsorbed solvent on the surface of FSM-16@Imine-Thiophen/Pd. Moreover, a weight loss between 200 and 480 °C of about 9% is relevant to the thermal decomposition of organic groups and complexes on the FSM-16 surface.

### 3.2. Synthesis of indeno[1,2,4]triazolo[1,5-*a*]pyrimidine

**Scheme 2:**
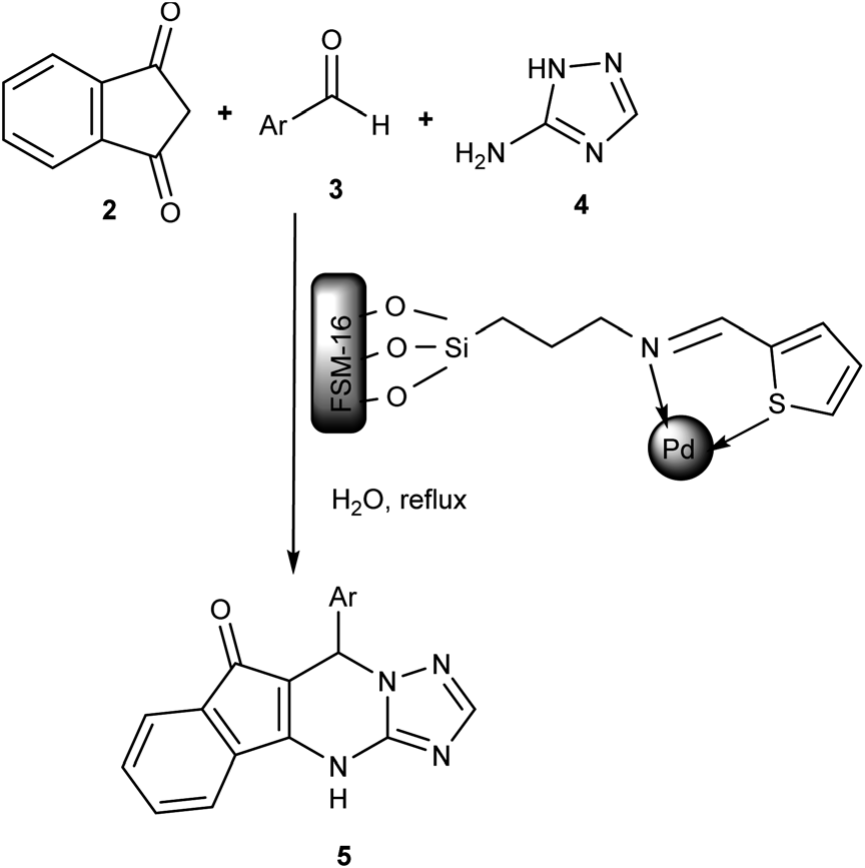
The synthesis of indeno[1,2,4]triazolo[1,5-*a*]pyrimidine derivatives in the presence of nanocatalyst 1.

After the successful synthesis and characterization of the FSM-16@Imine-Thiophen/Pd, its catalytic activity was investigated in the synthesis of indeno[1,2,4]triazolo[1,5-*a*]pyrimidine derivatives 5*via* the reaction between indan-1,3-dione 2, aromatic aldehydes 3 and 3-amino-1*H*-1,2,4-triazole 4 (Scheme 2).

Primarily, to optimize the reaction conditions, a reaction between benzaldehyde (1 mmol), indan-1,3-dione (1 mmol), and 3-amino-1*H*-1,2,4-triazole (1 mmol) was selected as the model reaction. Afterward, different parameters were studied, including various amounts of catalyst, a broad range of temperatures, and various solvents. Initially, the effect of the catalyst amount was examined, and accordingly, the best result was received in the attendance of 0.007 g of the FSM-16@Imine-Thiophen/Pd catalyst. The effect of the various solvents in the model reaction was investigated in the presence of solvents with different polarities and also under solvent-free conditions. The best reaction efficiency in H_2_O with higher polarity, as a green solvent, environmentally friendly, cheap and, available was obtained. However, due to the hydrophobic effect of organic compounds, the nature of the solvent can actually affect the reaction rate and order of a chemical reaction. Finally, the effect of temperature was also studied. Table 1 presents the summary of the details and results obtained from this study, in which the use of FSM-16@Imine-Thiophen/Pd (0.007 g) as the catalyst in H_2_O under reflux conditions will be the best condition. To investigate the generality of this protocol, the reaction of different aryl aldehydes, containing both electron-donating and electron-withdrawing groups, was employed in the reaction (Table 2). The reaction proceeded smoothly to afford the desired products 5 in good to excellent yields.

**Table 1:** The reaction condition optimization in the synthesis of 5a^a^

Entry	Catalyst 1 (g)	Solvent	Temp. (°C)	Yield^b^ (%)
1	—	—	25	—
2	—	—	80	5
3	—	—	90	8
4	—	—	100	12
5	0.003	—	100	40
6	0.005	—	100	65
7	0.007	—	100	95
8	0.008	—	100	95
9	0.007	EtOH	Reflux	75
10	0.007	EtOH : H_2_O	Reflux	80
11	0.007	MeOH	Reflux	70
12	0.007	H_2_O	Reflux	96
13	0.007	DMF	100	60
14	0.007	DMSO	100	70
15	0.007	Toluene	100	50
16	0.007	H_2_O	50	75
17	0.007	H_2_O	70	80
18	0.007	H_2_O	90	85

a Reaction conditions: benzaldehyde (1 mmol), 3-amino-1*H*-1,2,4-triazole (1 mmol), indan-1,3-dione (1 mmol), time: 80 min.

b Isolated yield.

**Table 2:** Synthesis of compound 5 using FSM-16@Imine-Thiophen/Pd^a^

Entry	Aldehyde	Product 5	Mp (°C)	Yield^b^ (%)
5a	C_6_H_5_CHO	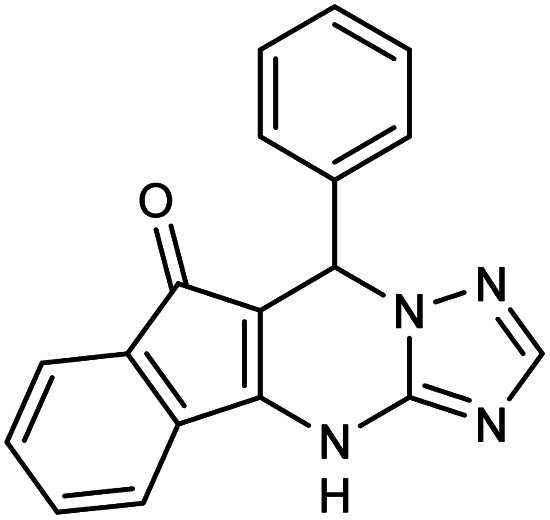	313–314	96
5b	4-Br–C_6_H_4_CHO	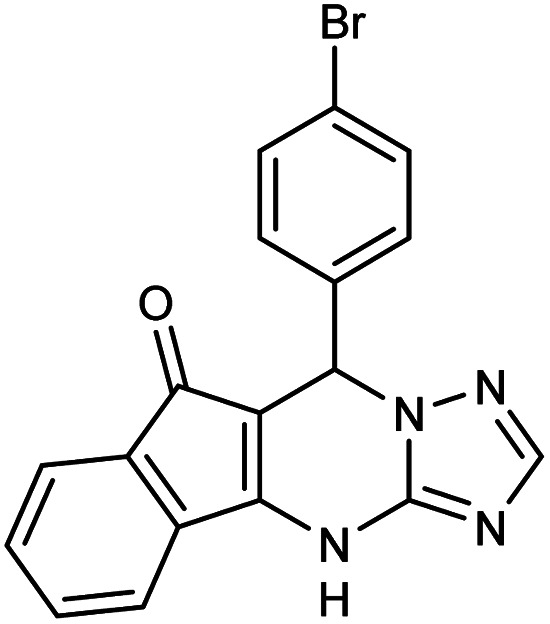	330–331	94
5b	4-Cl–C_6_H_4_CHO	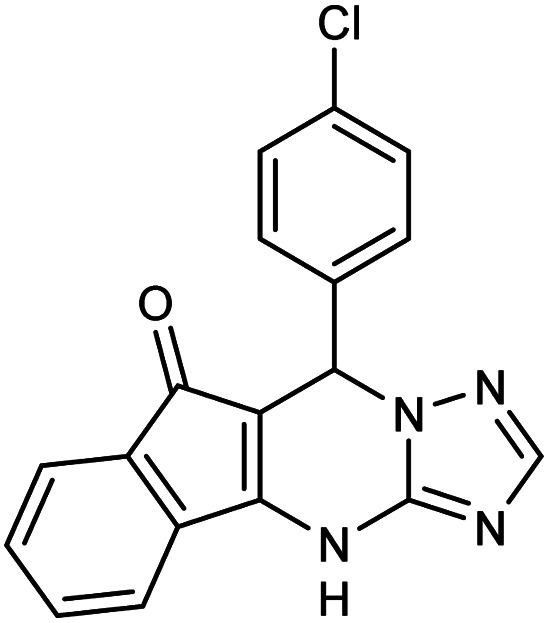	291–293	96
5d	4-NO_2_C_6_H_4_CHO	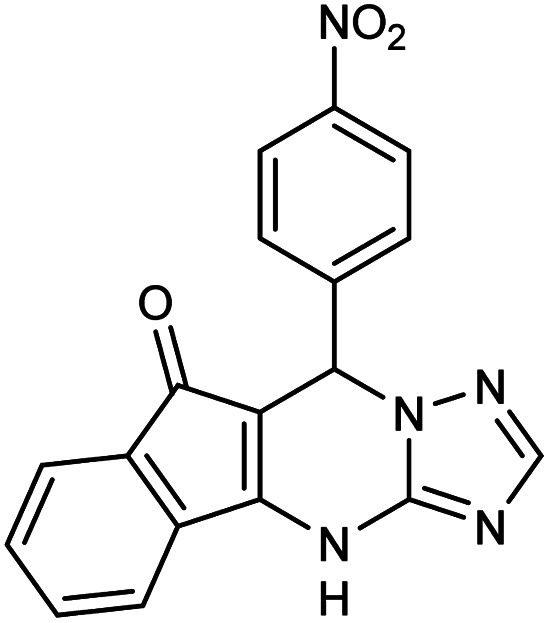	320–321	90
5e	Indole-3-carboxaldehyde	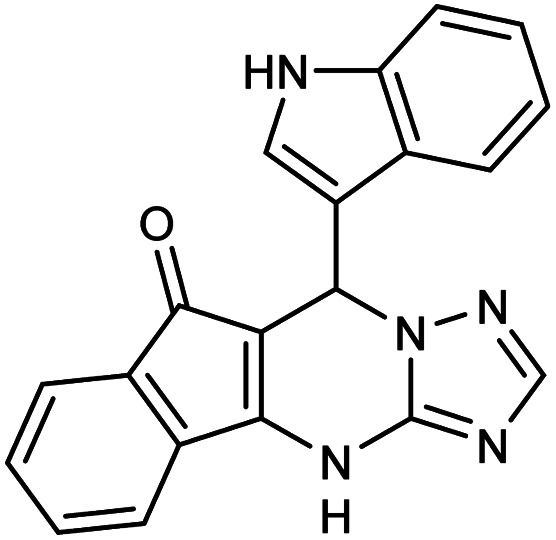	323–325	82
5f	4-PhCH_2_O–C_6_H_4_CHO	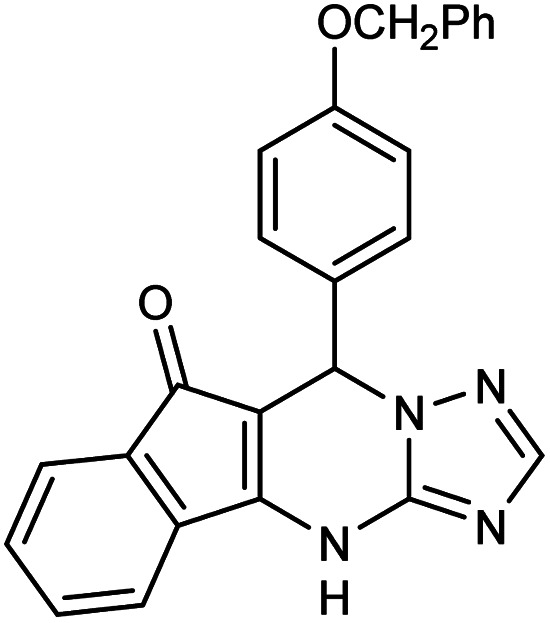	341–343	80
5g	2-CH_3_–C_6_H_4_CHO	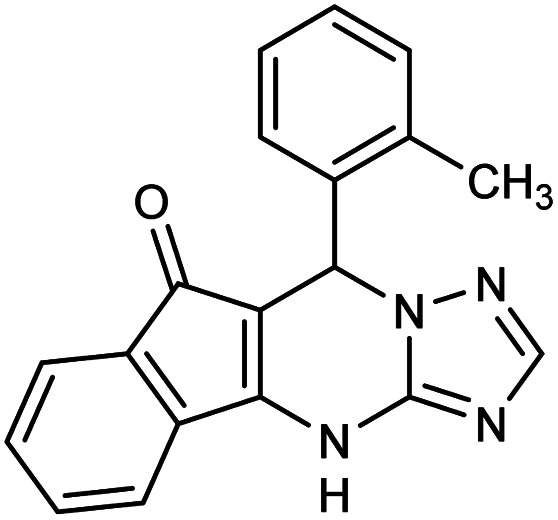	288–290	87
5h	3-OEt–4-OH–C_6_H_3_CHO	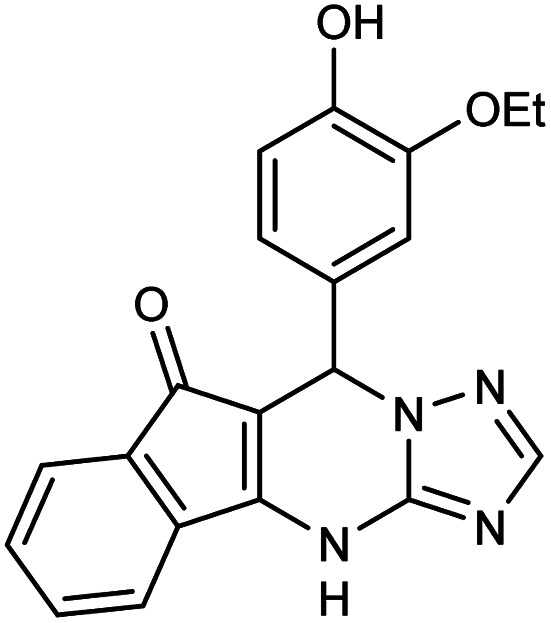	345–347	80

a Reaction conditions: aldehyde (1 mmol), 3-amino-1*H*-1,2,4-triazole (1 mmol), indan-1,3-dione (1 mmol) and FSM-16@Imine-Thiophen/Pd (0.007 g), H_2_O (5 mL), reflux conditions.

b Isolated yields.

**Scheme 3:**
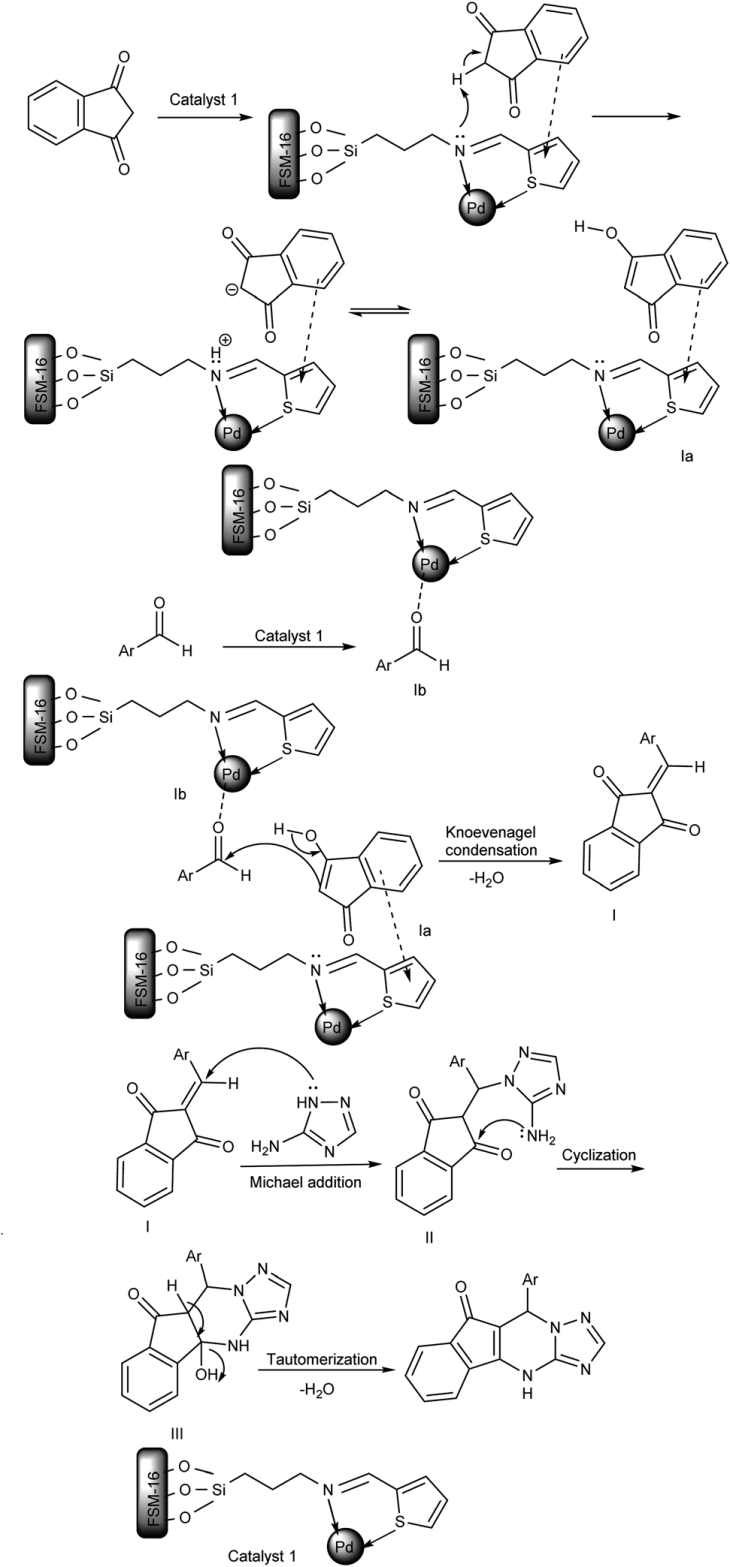
The suggested mechanism for synthesizing indeno[1,2,4]triazolo[1,5-*a*]pyrimidine derivatives using FSM-16@Imine-Thiophen/Pd.

The proposed mechanism for the preparation of indeno[1,2,4]triazolo[1,5-*a*]pyrimidine derivatives (5a–h) is shown in Scheme 3. Initially, benzene ring of indan-1,3-dione interacts with ring of thiophene *via* π-stacking and hydrogen atom located between two carbonyl groups shifts to N-atom with formation cation–anion pair which after enol transformation give intermediate (Ia). Next, aldehyde is activated by the Lewis acid site of the catalyst (intermediate Ib). Afterward, the activated carbonyl group of aldehyde (intermediate Ib) is attacked by active methylene of indan-1,3-dione (intermediate Ia) to produce intermediate I (Knoevenagel condensation). Next, 3-amino-1*H*-1,2,4-triazole attacks intermediate I to give II (Michael addition). Finally, the obtained intermediate III, using intramolecular cyclization and then tautomerization, provided the corresponding product 5 after losing H_2_O.

### 3.3. Efficiency of catalyst

**Fig. 8:**
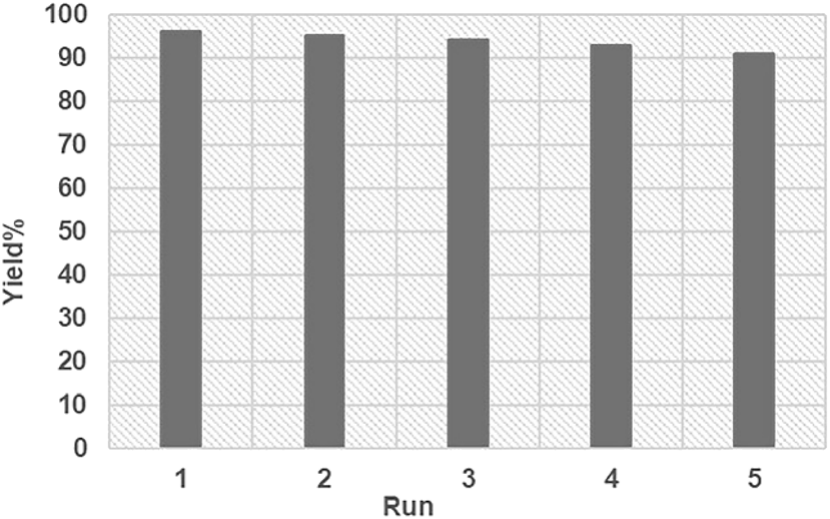
Reusability of FSM-16@Imine-Thiophen/Pd in the synthesis of 5a.

To investigate whether FSM-16@Imine-Thiophen/Pd operates in a homogeneous or heterogeneous manner, a filtration test has been done in the model reaction under optimized reaction conditions. After nearly 50% of the reaction progress, we separated the FSM-16@Imine-Thiophen/Pd catalyst, using filtration from the reaction mixture. Afterward, the mixture residue continued under optimal conditions, but no substantial increase in product conversion was observed. This experiment confirms that the nanocatalyst is completely heterogeneous, and no leaching of Pd occurs during the reaction process. To investigate the recyclability and reusability of FSM-16@Imine-Thiophen/Pd catalyst, the reaction of benzaldehyde, 3-amino-1*H*-1,2,4-triazole and indan-1,3-dione was performed under optimum reaction conditions. Once the reaction was complete, ethanol was added, and the catalyst was filtered and washed with ethanol. Next, the recovered catalyst was dried and used for five consecutive cycles without significant reduction in catalytic activity (Fig. 8).

**Fig. 9:**
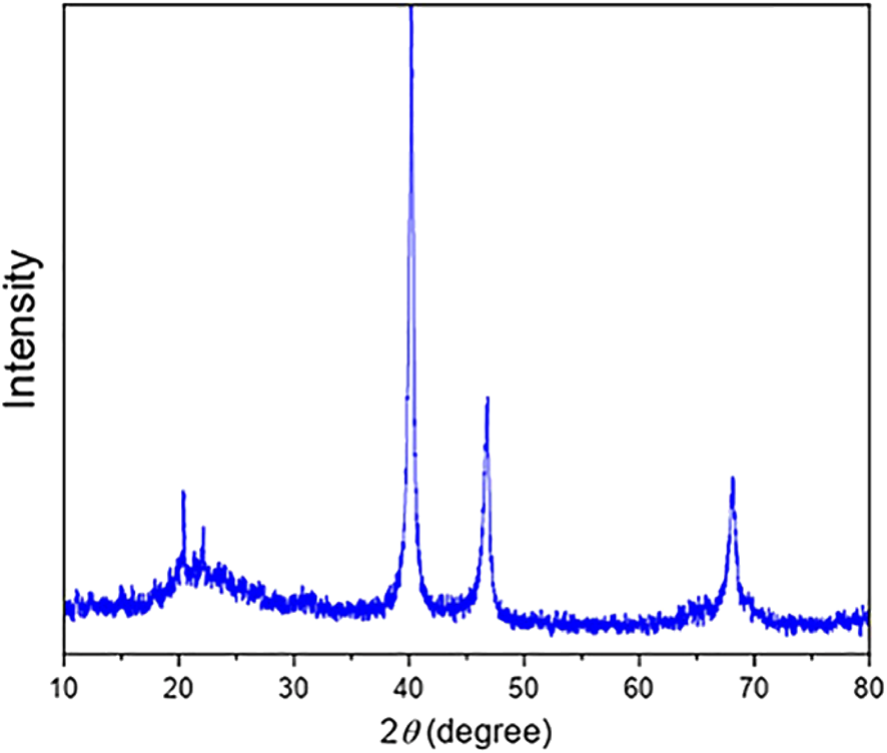
XRD pattern of the recycled FSM-16@Imine-Thiophen/Pd after the fifth reaction cycle.

**Fig. 10:**
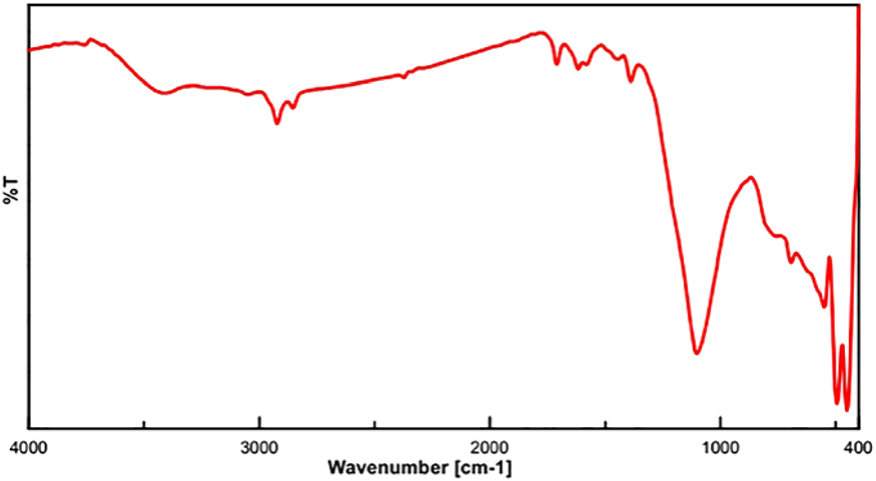
FT-IR spectrum of the recycled FSM-16@Imine-Thiophen/Pd nanocatalyst 1 (after five times).

The XRD diffraction patterns (Fig. 9) confirmed the position and relative intensity of the catalyst peaks after recycling and clearly showed their structural stability. Also, the FT-IR analysis was conducted to prove the stability of the catalyst structure following recycling. The results of this spectrum (Fig. 10) showed the high strength of the recycled catalyst.

## 4. Conclusions

In this article, a novel nanocatalyst based on FSM (FSM-16@Imine-Thiophen/Pd) was prepared and characterized. The XRD analysis, TEM and FE-SEM images of FSM-16@Imine-Thiophen/Pd demonstrated a highly uniform mesostructure. Furthermore, the FT-IR and TGA analyses confirmed the excellent stability of the immobilized palladium(ii) complex in the material network. FSM-16@Imine-Thiophen/Pd was successfully used as the catalyst to synthesize novel indeno[1,2,4]triazolo[1,5-*a*]pyrimidine derivatives. The nanocatalyst can be recovered from the reaction mixture using filtration and reused five times. Some specific advantages of this study are as follows: high yield of the products, using water as the reaction media, mild reaction conditions, short reaction times and high recyclability and stability of the catalyst.

## Conflicts of interest

There are no conflicts to declare.

## Supplementary Material

RA-012-D2RA06271B-s001
