# Supplementary material for: Synthesis and characterization of thiophene-derived palladium(ii) complex immobilized on FSM-16 and its application in the novel synthesis of 7-(aryl)-7,12-dihydro-6H-indeno[1,2,4]triazolo[1,5-a]pyrimidine-6-one derivatives
Source: RSC Adv. 2022 Nov 29;12(53):34325–34. doi: 10.1039/d2ra06271b (PMC9707470; doi:10.1039/d2ra06271b)

Electronic Supplementary Material (ESI) for RSC Advances.
This journal is © The Royal Society of Chemistry 2022

Supporting Information

Synthesis and characterization of thiophene-derived palladium (II) complex immobilized on FSM-16 and its application in the novel synthesis of 7-(aryl)-7,12-dihydro-6*H*-indeno [1,2,4]triazolo[1,5-*a*]pyrimidine-6-one derivatives

Azar Jahanbakhshi, Mahnaz Farahi*, Bahador Karami and Iman Sedighimehr

^{1}H NMR Spectra of Compound **5a**

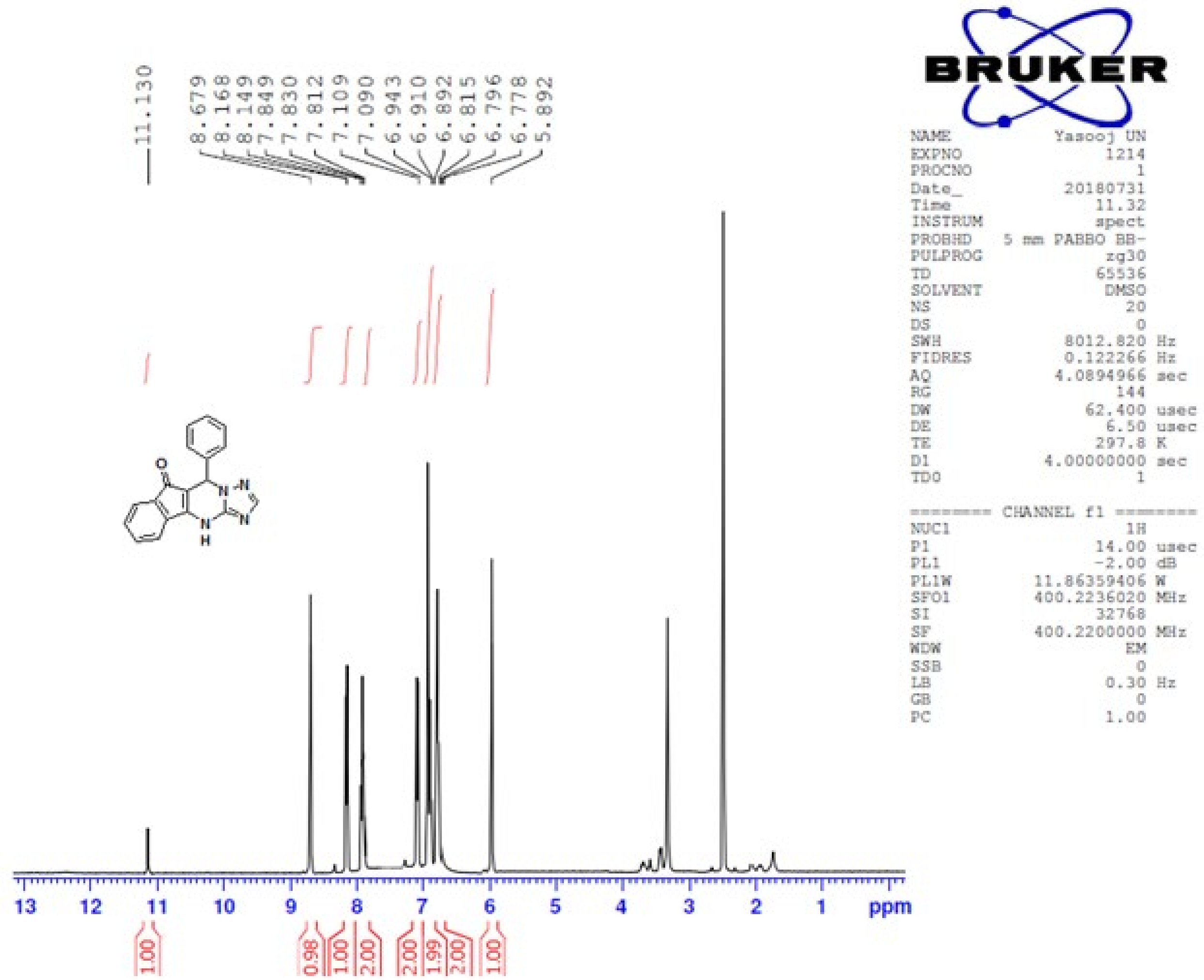

^{13}C NMR Spectra of Compound **5a**

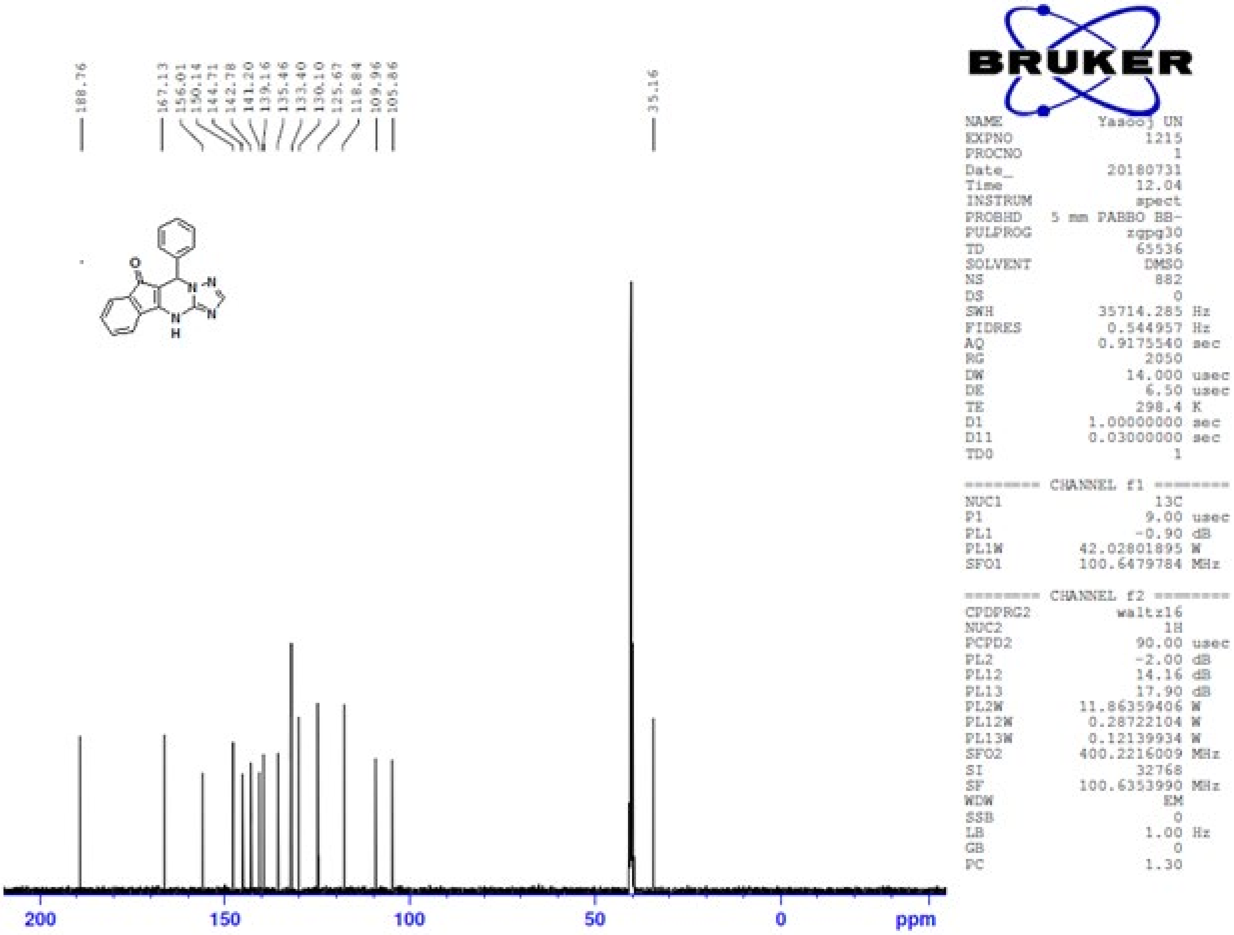

^{1}H NMR Spectra of Compound **5b**

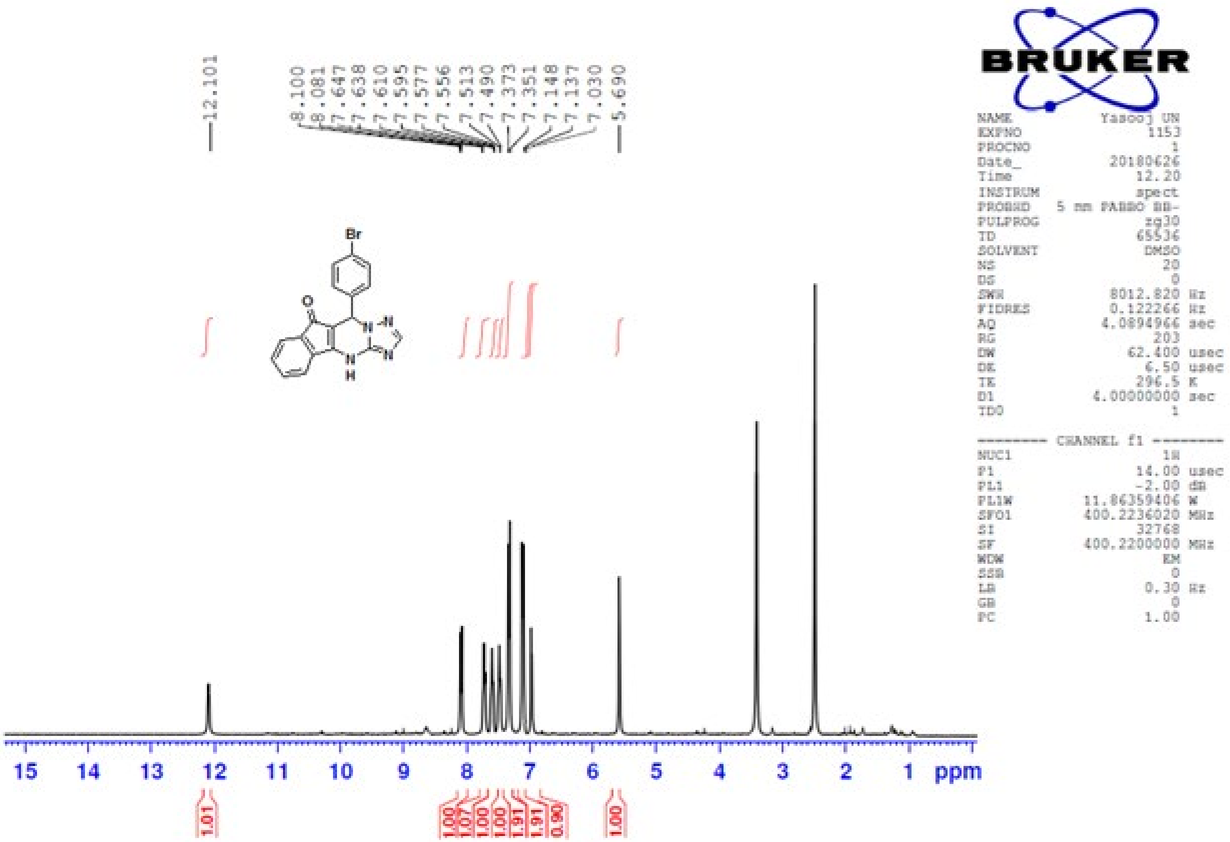

^{13}C NMR Spectra of Compound **5b**

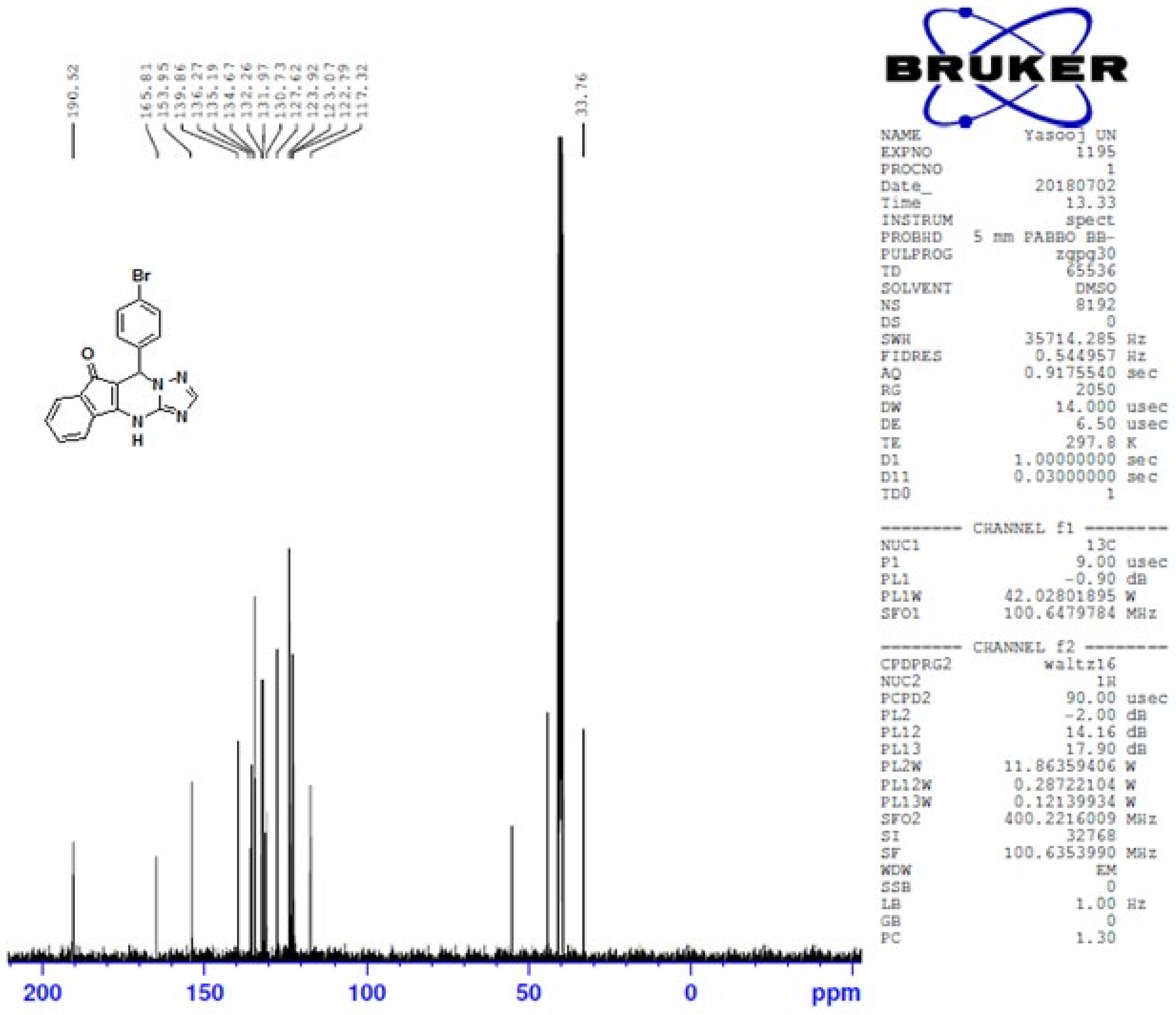

^{1}H NMR Spectra of Compound **5d**

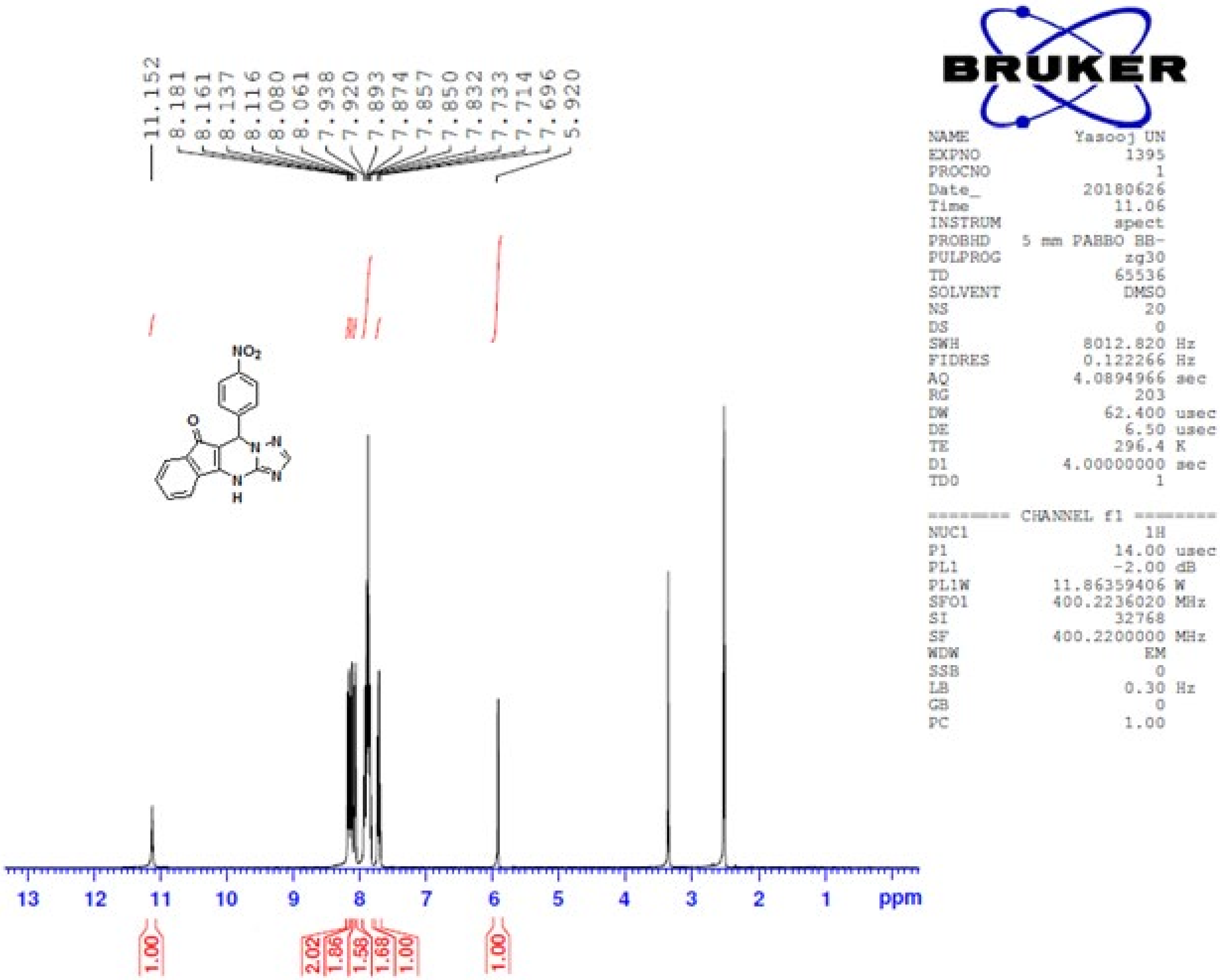

^{13}C NMR Spectra of Compound **5d**

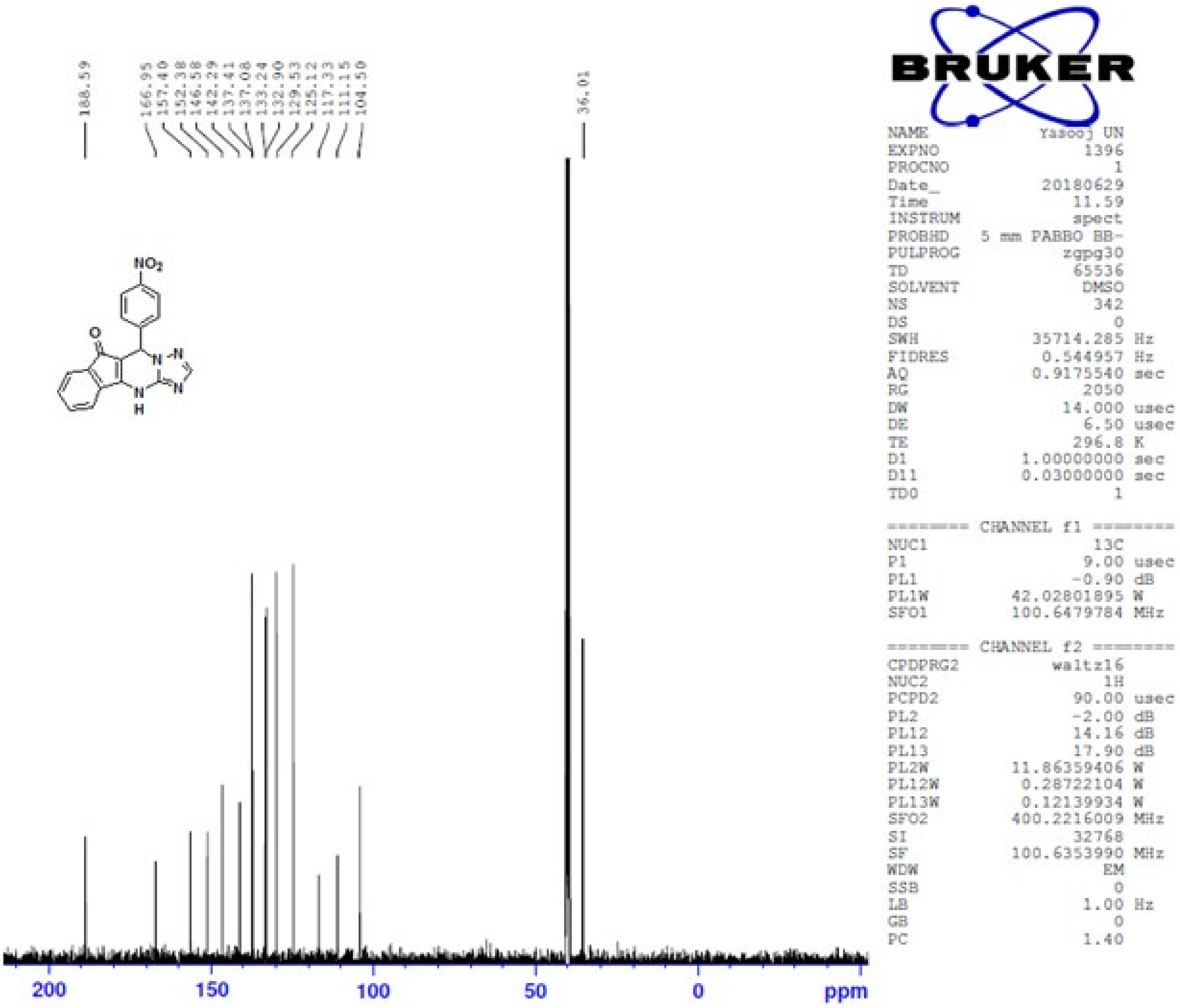

^{1}H NMR Spectra of Compound **5e**

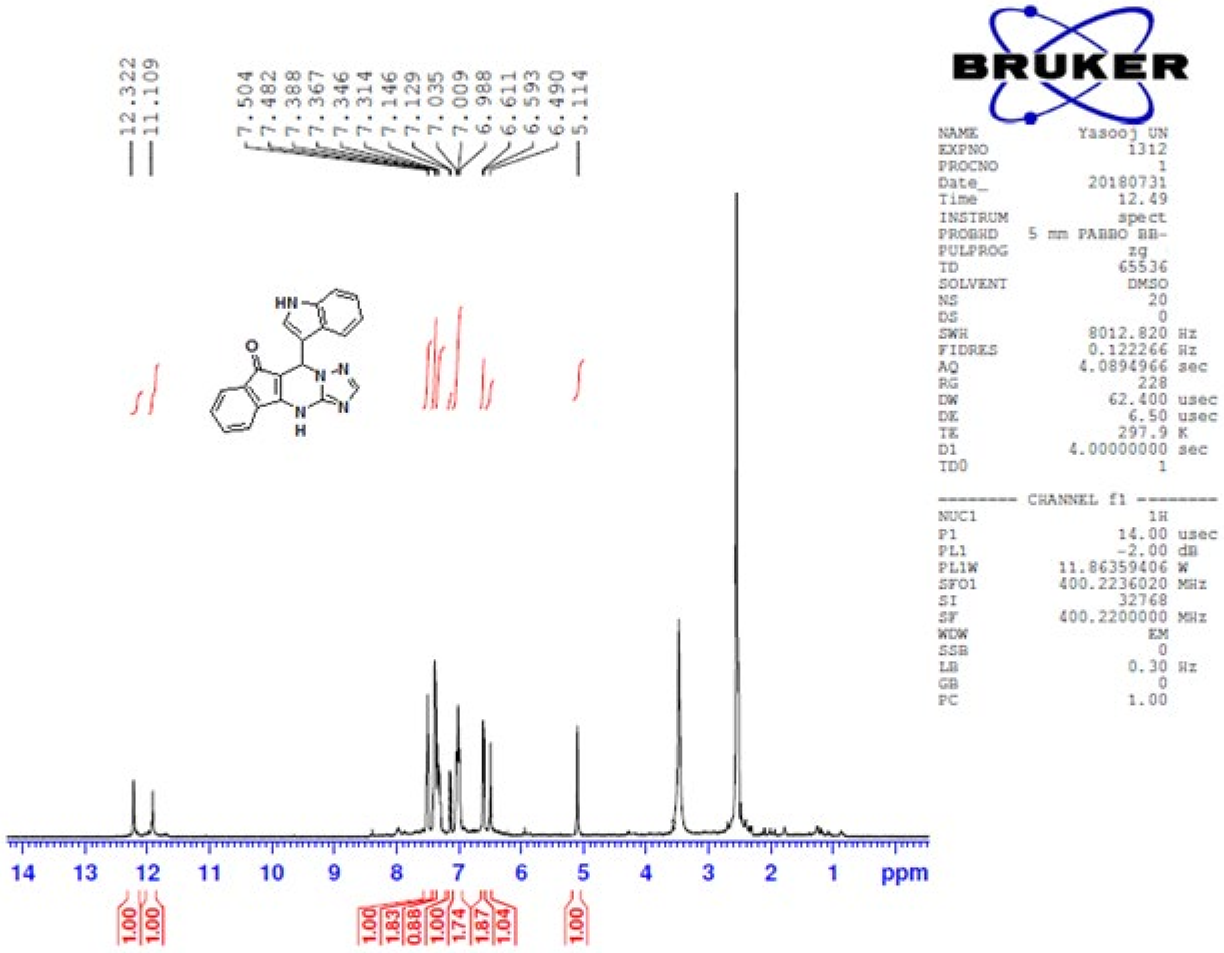

^{13}C NMR Spectra of Compound **5e**

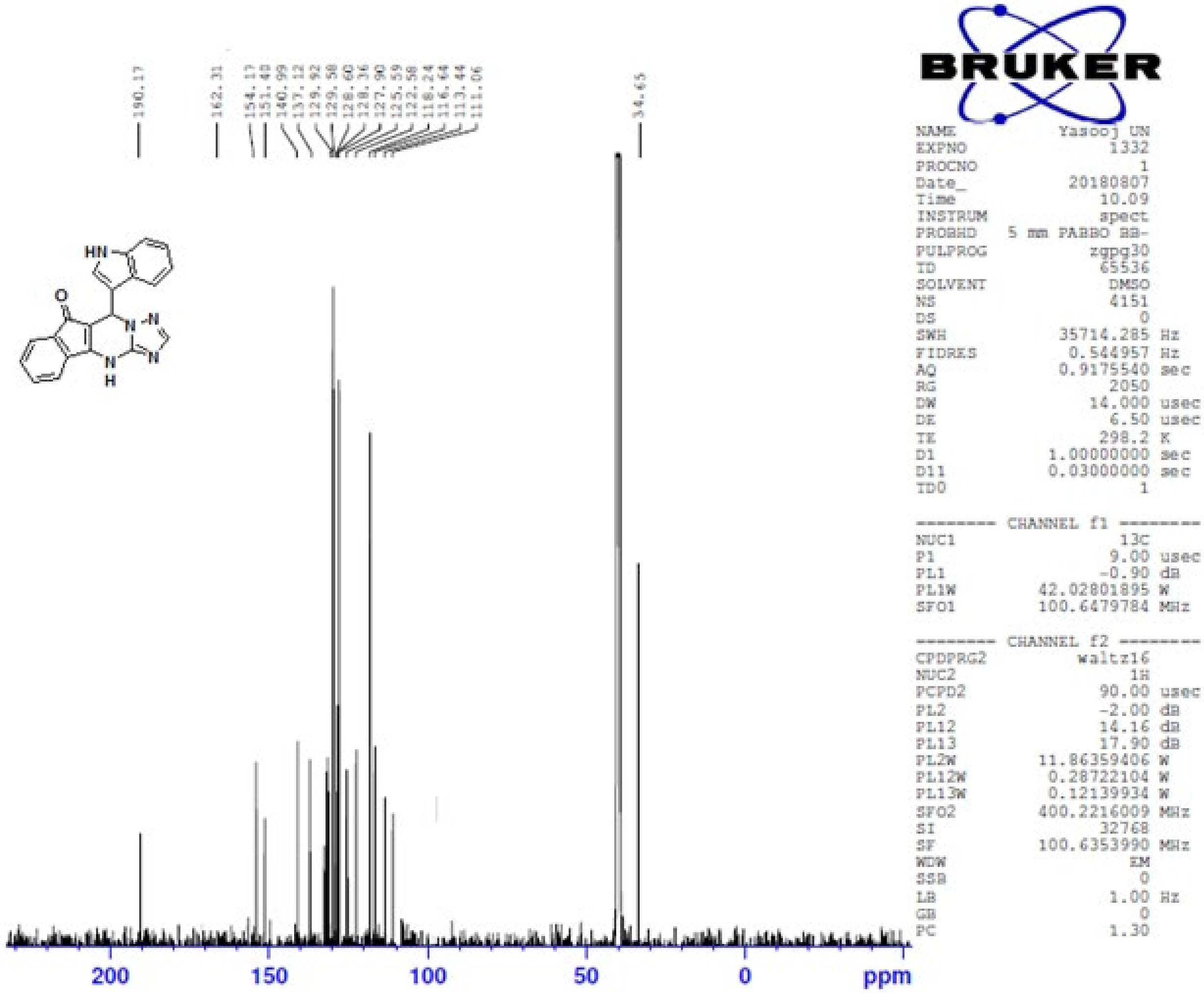

^{1}H NMR Spectra of Compound **5h**

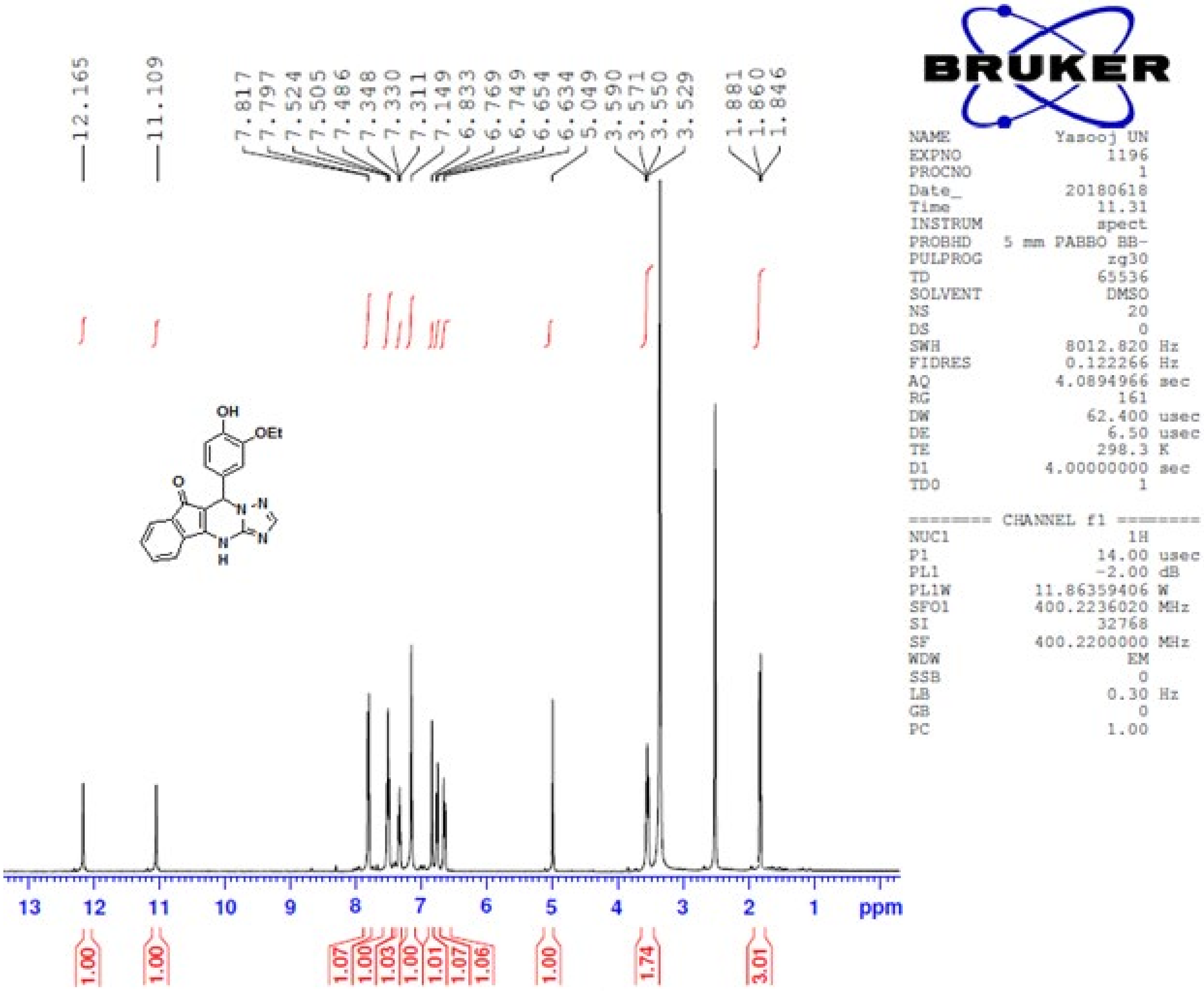

^{13}C NMR Spectra of Compound **5h**

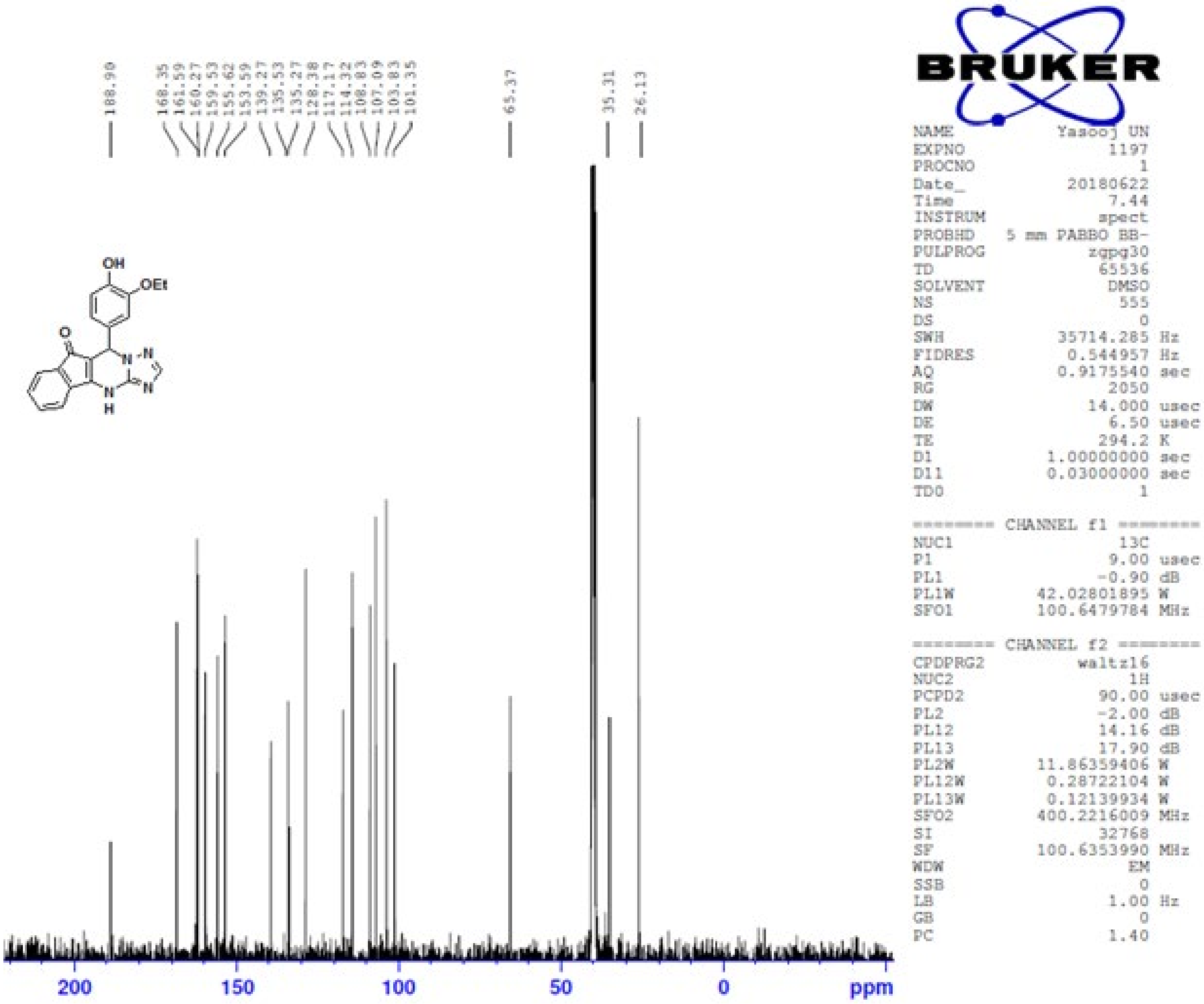

Supplement: RA-012-D2RA06271B-s001 [file RA-012-D2RA06271B-s001.pdf]
